# Genetic variation in the *Catechol-O-Methyltransferase (COMT) *gene and morphine requirements in cancer patients with pain

**DOI:** 10.1186/1744-8069-4-64

**Published:** 2008-12-18

**Authors:** Trude T Rakvåg, Joy R Ross, Hiroe Sato, Frank Skorpen, Stein Kaasa, Pål Klepstad

**Affiliations:** 1 Department of Cancer Research and Molecular Medicine, Faculty of Medicine, Norwegian University of Science and Technology (NTNU), N-7489 Trondheim, Norway; 2 St Joseph's Hospice, London, UK; 3 Clinical Genomics Group, Imperial College, London, UK; 4 Department of Laboratory Medicine Children's and Women's Health, NTNU, N-7489 Trondheim, Norway; 5 Department of Oncology and Radiotherapy, St. Olavs Hospital, Trondheim, Norway; 6 Department of Circulation and Medical imaging, NTNU, N-7489 Trondheim, Norway; 7 Department of Intensive Care Medicine, St. Olavs Hospital, Trondheim, Norway

## Abstract

**Background:**

Genetic variation contributes to differences in pain sensitivity and response to different analgesics. Catecholamines are involved in the modulation of pain and are partly metabolized by the catechol-O-methyltransferase (COMT) enzyme. Genetic variability in the *COMT *gene may therefore contribute to differences in pain sensitivity and response to analgesics. It is shown that a polymorphism in the *COMT *gene, Rs4680 (Val158Met), influence pain sensitivity in human experimental pain and the efficacy for morphine in cancer pain treatment. In this study we wanted to investigate if variability in other regions in the *COMT *gene also contributes to interindividual variability in morphine efficacy.

**Results:**

We genotyped 11 single nucleotide polymorphisms (SNPs) throughout the *COMT *gene, and constructed haplotypes from these 11 SNPs, which were in Hardy-Weinberg equilibrium. We compared both genotypes and haplotypes against pharmacological, demographical and patient symptoms measurements in a Caucasian cancer patient cohort (n = 197) receiving oral morphine treatment for cancer pain. There were two frequent haplotypes (34.5% and 17.8%) in our cohort. Multivariate analyses showed that patients carrying the most frequent haplotype (34.5%) needed lower morphine doses than patients not carrying the haplotype, with a reduction factor of 0.71 (p = 0.005). On the allele level, carriers of alleles for six of the SNPs show weak associations in respect to morphine dose and the alleles associated with the lowest morphine doses constitute part of the most frequent haplotype.

**Conclusion:**

This study suggests that genetic variability in the *COMT *gene influence the efficacy of morphine in cancer patients with pain, and that increased understanding of this variability is reached by expanding from analyses of single SNPs to haplotype construction and analyses.

## Background

One of the genes in which variability is believed to contribute to differences in pain sensitivity and response to analgesics is the *catechol-O-methyltransferase *(*COMT*) gene [1-3]. The COMT enzyme metabolises catecholamines such as dopamine, noradrenaline and adrenaline. The most studied single nucleotide polymorphism (SNP) in the *COMT *gene is the Rs4680, also known as Val158Met. This polymorphism causes a substitution from a valine (Val) to a methionine (Met) at amino acid position 158, leading to a three- to four-fold reduced activity of the COMT enzyme [4]. Because of the influence on COMT activity by the Rs4680 (Val158Met) SNP and the well established involvement of catecholamines in pain perception [5-7], several studies have investigated if this SNP can explain interindividual variability in pain perception and efficacy of analgesics. Zubieta *et al*., demonstrated that individuals with the Met/Met genotype had higher sensory and affective ratings of pain and a higher regional density of mu opioid receptors in the brain [1]. The Rs4680 (Val158Met) SNP has also been shown to influence efficacy of morphine used for cancer pain, for which the Met/Met genotype group needed lower morphine doses than Val/Val genotype group [2]. Results from these two studies are intriguing since individuals with the Met/Met genotype report higher pain ratings, but need less morphine. However, as authors discuss [2], the increase of mu opioid receptor density seen in Met/Met genotype individuals [1], may explain why morphine is more effective in individuals carrying this genotype.

Other researchers have investigated other SNPs across the *COMT *gene and shown that other regions of the gene may also contribute to pain perception [3,8] and influence morphine-related side-effects [9]. Diatchenko *et al*., identified three genetic variants (haplotypes) in the *COMT *gene and designated them as low pain sensitivity (LPS), average pain sensitivity (APS) and high pain sensitivity (HPS) haplotypes. The Rs4680 (Val158Met) polymorphism was one of four SNPs included in their haplotype analyses. The authors argue that the Rs4680 (Val158Met) SNP cannot account for the observed variations in pain perception alone, since both the LPS and HPS haplotypes possess the G allele that codes for the more stable Val variant of the COMT enzyme [3]. Kim *et al*., analysed 13 SNPs in the *COMT *gene and their association to acute post-surgical pain in humans [8]. The authors found that the Rs740603 polymorphism showed significant association with maximum post-operative pain rating, but did not observe any association between other SNPs, including the Rs4680 (Val158Met) SNP, and pain score. Ross *et al*., found that a SNP in intron 1 (Rs740603) and a haplotype, defined by SNPs in the promoter region and intron 1, were significantly associated with drowsiness and confusion or hallucinations in a cancer patient cohort treated with morphine. In the study by Ross *et al*., the Rs4680 (Val158Met) SNP did not influence the risk for morphine induced adverse effect [9].

All the three studies cited above that have investigated multiple SNPs in the *COMT *gene have either pain perception or the risk for opioid adverse effects as the primary endpoint in the study [3,8,9]. No studies have investigated if other SNPs than the Rs4680 (Val158Met) in the *COMT *gene are important for the analgesic efficacy of morphine. Therefore, in a patient cohort in which we have previously shown that the Rs4680 (Val158Met) polymorphism influences the efficacy of morphine for cancer pain [2], we investigated if variability in other regions in the *COMT *gene also contribute to interindividual variability in morphine efficacy. In addition to examining the effect from each individual SNP we constructed long haplotypes in order to study composite effect from combinations of 11 SNPs along the gene.

## Results

DNA from 197 patients receiving oral morphine treatment for cancer pain was analysed in this study.

### Genotype and haplotype distribution

**Figure 1:**
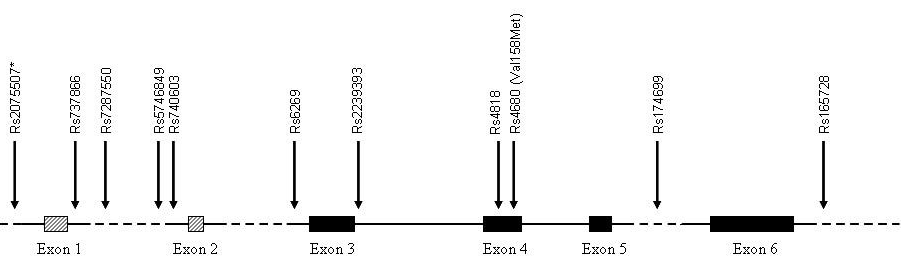
**Schematic diagram of the *COMT *gene**. Schematic diagram of the *catechol-O-methyltransferase *(*COMT*) gene, labeled with the 11 SNPs analysed in the present study. *Rs2075507 has recently been revised, the former SNP number was Rs2097603. Exon 1 and exon 2 are non-coding.

A schematic presentation of the 11 SNPs analysed in the *COMT *gene is shown in Figure 1. The genotype frequencies, allele frequencies and allele carriage for all 11 SNPs analysed are shown in Table 1. All SNPs were in Hardy-Weinberg equilibrium. The long haplotypes constructed from the 11 SNPs in the *COMT *gene are shown in Table 2. The frequencies of the two most common haplotypes were 34.5% and 17.8%. Fourteen different haplotypes with a frequency of > 1% described 91% of the population. We designated the haplotypes as haplotype 1 to haplotype 14, corresponding to the frequency at which they occur; haplotype 1 being the most frequent.

**Table 1:** *Catechol-O-methyltransferase *(*COMT*) genotype frequencies, allele frequencies and allele carriage in the total of 197 cancer patients

SNP (region)	Genotype	Genotype frequencies	Allele	Allele frequencies	Allele carriage
Rs2075507*	AA	0.26	A	0.53	0.80
(promoter)	AG	0.54	G	0.47	0.74
	GG	0.20			

Rs737866	AA	0.61	A	0.78	0.94
(intron 1)	AG	0.33	G	0.22	0.39
	GG	0.06			

Rs7287550	CC	0.53	C	0.72	0.91
(intron 1)	CT	0.38	T	0.28	0.47
	TT	0.09			

Rs5746849	GG	0.18	G	0.43	0.68
(intron 1)	GA	0.49	A	0.57	0.82
	AA	0.33			

Rs740603	AA	0.31	A	0.56	0.81
(intron 1)	AG	0.50	G	0.44	0.69
	GG	0.19			

Rs6269	AA	0.40	A	0.62	0.84
(intron 2)	AG	0.44	G	0.38	0.60
	GG	0.16			

Rs2239393	AA	0.40	A	0.62	0.84
(intron 3)	AG	0.44	G	0.38	0.59
	GG	0.16			

Rs4818	CC	0.41	C	0.63	0.84
(exon 4)	CG	0.43	G	0.37	0.59
	GG	0.16			

Rs4680 (Val158Met)	GG	0.22	G	0.44	0.66
(exon 4)	GA	0.44	A	0.56	0.78
	AA	0.34			

Rs174699	CT	0.09	C	0.04	0.09
(intron 5)	TT	0.91	T	0.96	100.0

Rs165728	CT	0.10	C	0.05	0.10
(untranslated region)	TT	0.90	T	0.95	100.0

* Rs2075507 has recently been revised, earlier SNP number was Rs2097603

**Table 2:** COMT haplotype frequencies.

	SNP position (5' to 3')											Haplotype frequency
Haplotype	Rs 2075507	Rs 737866	Rs 7287550	Rs 5746849	Rs 740603	Rs 6269	Rs 2239393	Rs 4818	Rs 4680 (Val158Met)	Rs 174699	Rs 165728	n chromosomes (%)

1	G	A	C	A	A	A	A	C	A	T	T	136 (34.5)
2	A	G	C	G	G	G	G	G	G	T	T	70 (17.8)
3	A	A	T	G	G	A	A	C	A	T	T	33 (8.4)
4	A	A	T	G	G	G	G	G	G	T	T	24 (6.1)
5	G	A	C	A	A	G	G	G	G	T	T	18 (4.6)
6	A	A	T	A	A	A	A	C	A	T	T	18 (4.6)
7	G	A	C	G	G	G	G	G	G	T	T	10 (2.5)
8	A	A	C	A	A	G	G	G	G	T	T	9 (2.3)
9	A	A	T	G	G	A	A	C	G	T	T	9 (2.3)
10	A	A	C	A	A	A	A	C	A	T	T	9 (2.3)
11	A	A	T	A	A	A	A	C	G	C	C	8 (2.0)
12	A	A	T	A	G	A	A	C	A	T	T	6 (1.5)
13	G	A	T	A	A	G	G	C	G	T	T	4 (1.0)
14	G	G	C	A	A	A	A	C	A	T	T	4 (1.0)
X	-	-	-	-	-	-	-	-	-	-	-	36 (9.1)

*Catechol-O-methyltransferase *(*COMT*) haplotype frequencies for the total of 197 cancer patients.

### Morphine dose and genotypes

The pharmacological observations for genotype groups and allele carriage are shown in Table 3. The median morphine dose requirements between genotype groups for the Rs4818 polymorphism were 60, 80 and 120 mg/24 h for the CC, CG and GG genotype groups, respectively (p = 0.042) and for the Rs4680 (Val158Met) polymorphism the median morphine doses were 90, 80 and 60 mg/24 h for the GG, GA and AA genotype groups, respectively (p = 0.022). For six of the SNPs (Rs5746849, Rs740603 in intron 1, Rs6269 in intron 2, Rs2239393 in intron 3 and Rs4818 and Rs4680 (Val158Met) in exon 4) allele carriers showed a tendency to differences in median morphine doses. (Table 3).

**Table 3:** Pharmacological observations.

SNP	Genotype	Morphine dose mg/24 h median [range]	P value	Allele carriage	Morphine dose mg/24 h Median [range]	P value
Rs2075507	AA	90 [20–420]	0.220	A	80 [20–760]	0.90
	AG	70 [20-480]		Not A	60 [10–660]	
	GG	60 [10–660]		G	70 [10–760]	0.09
				Not G	90 [20–420]	

Rs737866	AA	70 [10–660]	0.486	A	73 [10–760]	0.26
	AG	80 [20–760]		Not A	90 [20–350]	
	GG	90 [20–350]		G	80 [20–760]	0.45
				Not G	70 [10–660]	

Rs7287550	CC	70 [20–760]	0.862	C	78 [10–760]	0.59
	CT	80 [10–410]		Not C	120 [30–420]	
	TT	120 [30–420]		T	80 [10–420]	0.91
				Not T	70 [20–760]	

Rs5746849	GG	115 [20–420]	0.103	G	80 [20–420]	0.57
	GA	80 [20–390]		Not G	65 [10–760]	
	AA	65 [10–760]		A	70 [10–760]	0.03^c^
				Not A	115 [20–420]	

Rs740603	AA	70 [10–760]	0.099	A	70 [10–760]	0.04^c^
	AG	80 [20–390]		Not A	110 [20–420]	
	GG	110 [20–420]		G	80 [20–420]	0.92
				Not G	70 [10–760]	

Rs6269	AA	70 [20–660]	0.090	A	70 [10–660]	0.03^c^
	AG	75 [10–480]		Not A	120 [20–760]	
	GG	120 [20–760]		G	80 [10–760]	0.32
				Not G	70 [20–660]	

Rs2239393	AA	70 [20–660]	0.093	A	70 [10–660]	0.03^c^
	AG	73 [10–480]		Not A	120 [20–760]	
	GG	120 [20–760]		G	80 [10–760]	0.42
				Not G	70 [20–660]	

Rs4818	CC	60 [10–660]	0.042 ^a^	C	70 [10–660]	0.04^c^
	CG	80 [20–480]		Not C	120 [20–760]	
	GG	120 [20–760]		G	80 [20–760]	0.04^c^
				Not G	60 [10–660]	

Rs4680 (Val158Met)	GG	90 [20–760]	0.022 ^b^	G	80 [10–760]	0.045^c^
	GA	80 [10–480]		Not G	60 [20–660]	
	AA	60 [20–660]		A	70 [10–660]	0.07
				Not A	90 [20–760]	

Rs174699	CT	80 [20–480]	0.666	C	80 [20–480]	0.67
				Not C	73 [10–760]	
	TT	73 [10–760]		T	80 [10–760]	-
				Not T	-	

Rs165728	CT	80 [20–480]	0.457	C	80 [20–480]	0.46
				Not C	70 [10–760]	
	TT	70 [10–760]		T	80 [10–760]	-
				Not T	-	

a Kruskal-Wallis test for 3 independent samples; b Jonckheere Tepstra test for 3 independent samples; c Mann-Whitney U test for 2 independent samples.

Pharmacological observations for genotype groups and allele carriage.

### Morphine dose and haplotypes

We observed that carriers of haplotype 1, the most frequent haplotype in this Caucasian population (Table 2), needed less morphine than non-carriers, with a median morphine dose of 60 mg/24 h for carriers versus 100 mg/24 h for non-carriers (p = 0.006) (Table 4a). The serum concentrations of morphine, morphine-6-glucuoride (M6G) and morphine-3-glucuronide (M3G) reflected the different morphine doses between haplotypes, but no differences were statistically significant (Table 4b).

**Table 4:** Pharmacological observations **I** % **II**

**a – Pharmacological observations I**. Morphine dose and haplotype groups					
Haplotype	Carriage	N	Morphine dose *mg/24 h *median [range]	P value	

1	Yes	114	60 [10–660]	0.006 ^a^	
	No	83	100 [20–760]		

2	Yes	61	80 [20–410]	0.94	
	No	136	70 [10–760]		

3	Yes	31	90 [20–420]	0.44	
	No	166	78 [10–760]		

4	Yes	24	70 [20–420]	0.94	
	No	173	80 [10–760]		

5	Yes	17	120 [20–760]	0.17	
	No	180	78 [10–660]		

6	Yes	17	120 [30–200]	0.56	
	No	180	73 [10–760]		

7	Yes	10	120 [40–290]	0.44	
	No	187	80 [10–760]		

N = number of patients a Mann-Whitney U test for independent samples.					

**b – Pharmacological observations II**. Serum concentration of morphine, M6G and M3G against haplotype groups					

Haplotype	Carriage	N	Morphine (*nmol/ml*) median [range]	M6G (*nmol/ml*) median [range]	M3G (*nmol/ml*) median [range]

1	Yes	114	51 [2–350]	310 [10–2660]	1810 [120–16200]
	No	83	59 [3–1070]	349 [20–4830]	2310 [110–21250]

2	Yes	61	50 [3–330]	346 [20–2482]	1890 [120–12390]
	No	136	60 [2–1070]	319 [10–4830]	2040 [110–21250]

3	Yes	31	67 [5–320]	310 [29–1690]	2200 [197–7780]
	No	166	50 [2–1070]	330 [10–4830]	1992 [110–21250]

4	Yes	24	51 [3–277]	380 [20–2482]	2213 [110–12390]
	No	173	57 [2–1070]	320 [10–4830]	1992 [120–21250]

5	Yes	17	80 [4–1070]	403 [20–4830]	2415 [110–21250]
	No	180	52 [2–519]	325 [10–2660]	1960 [120–16200]

6	Yes	17	90 [9–230]	470 [120–1105]	3118 [1017–5481]
	No	180	50 [2–1070]	318 [10–4830]	1890 [110–21250]

7	Yes	10	50 [6–220]	460 [81–1809]	1832 [490–9460]
	No	187	58 [2–1070]	330 [10–4830]	2020 [110–21250]

N = number of patients; M6G = Morphine-6-glucuronide; M3G = Morphine-3-glucuronide

No statistical differences between carriers and non-carriers of the different haplotype groups for morphine, M6G and M3G serum concentration

Patient symptoms including average pain, fatigue, nausea and vomiting, dyspnea, sleep, appetite, constipation and cognitive function were similar for carriers and non-carriers of haplotype 1 (Table 5). Also the patient characteristics age, gender, tumour diagnosis, performance status, creatinine and albumin serum concentration, time since morphine treatment started and survival time after study were similar between the two genetic groups (Table 6). We observed that the carriers of haplotype 1 have had the cancer diagnosis longer (45 months) than non-carriers of haplotype 1 (31 months) (p = 0.03; Table 6). However there were no differences in time since morphine treatment started between carriers and non-carriers of haplotype 1 (3.4 and 3.6 months respectively; p = 0.47).

**Table 5:** Patient symptoms.

	Haplotype 1		P value ^a^
	Carriers	Non-carriers	
BPI average pain	3.5 (2.6)	3.9 (2.2)	0.26
Fatigue (EORTC score)	64.5 (23.5)	68.6 (23.1)	0.28
Nausea and vomiting (EORTC score)	26.6 (25.9)	27.0 (28.5)	0.77
Dyspnea (EORTC score)	36.6 (32.5)	34.4 (34.7)	0.80
Sleep (EORTC score)	35.3 (36.0)	32.8 (35.2)	0.58
Appetite (EORTC score)	53.2 (37.6)	54.3 (37.2)	0.95
Constipation (EORTC score)	54.5 (37.9)	55.7 (38.4)	0.77
Mini mental examination sum score	26.1 (3.4)	25.6 (4.0)	0.66

a Mann-Whitney U test for 2 independent samples

Patient symptoms for carriers and non-carriers of haplotype 1.

**Table 6:** Patient demographics.

	Haplotype 1		P value
	Carriers	Non-carriers	
Age	63 (13)	64 (12)	0.68
Gender:			
Male	68 (60%)	44 (53%)	0.38
Female	46 (40%)	39 (47%)	
Tumour diagnosis:			
Urological	38	19	0.80
Lung	20	17	
Breast	25	19	
Gastrointestinal	7	10	
Haematological	10	6	
Others	14	12	
Karnofsky performance status	67 (14)	66 (13)	0.36
Creatinine serum (μmol/l)	86 (28)	87 (39)	0.48
Albumin serum (g/l)	33 (5)	32 (5)	0.12
Time since diagnosis (months)	45 (52)	31 (43)	0.03^a^
Time since morphine treatment started (months)	3.4 (7.8)	3.6 (5.9)	0.47
Survival time after study (months)	5.7 (6.2)	4.8 (5.5)	0.23

Numbers in the table are given as mean (SD) or absolute numbers (%)

a Mann-Whitney U test for 2 independent samples

Patient demographics for carriers and non-carriers of haplotype 1.

In a multivariate stepwise linear regression analysis the variables "time since morphine treatment started" and haplotype 1 were shown to influence the morphine dose (p = 0.001 and p = 0.005; Table 7). After adjusting for the variable "time since morphine treatment started", the carriers of haplotype 1 still require lower morphine doses than patients that do not carry haplotype 1. Time since morphine treatment started is positively associated to morphine dose, whereas the carriers of haplotype 1 is predicted to need lower doses of morphine than non-carriers of haplotype 1 with a reduction factor of 0.71 (see discussion for calculation).

**Table 7:** Regression analysis. Morphine dose regression analysis

	b	SE	P value
Haplotype 1	-0.147	0.051	0.005
Time since morphine treatment started	0.013	0.004	0.001
Constant	1.95	0.042	

The logarithm (log_10_) of the 24 hour oral morphine dose was the dependent variable in this regression analysis. The regression coefficient, b, is an estimate of the parameter beta. SE = standard errors

## Discussion

We have identified a frequent haplotype (haplotype 1) in the *COMT *gene that may influence the morphine dose requirements in cancer patients with pain. Patients who carry haplotype 1 need lower morphine doses to relieve pain than patients that do not carry this haplotype (p = 0.006). The carriers of haplotype 1 are also carriers of the A allele for the Rs4680 (Val158Met) polymorphism, which is in agreement with our earlier observation that carriers of the Met variant of the enzyme (= A allele) need lower morphine doses than carriers of the Val variant of the COMT enzyme [2]. However, the effect of the A allele for the Rs4680 (Val158Met) polymorphism is not seen for haplotype 3 (Table 4a).

The Rs4680 (Val158Met) polymorphism is the most studied SNP in the *COMT *gene because the valine (Val) to methionine (Met) substitution leads to a three-to four-fold reduced activity of the COMT enzyme [4], hence the Val/Val, Val/Met and Met/Met genotypes predict a high, intermediate and low COMT enzyme activity, respectively. As the COMT enzyme metabolises catecholamines, a low COMT enzyme activity could result in an enhanced activation of dopaminergic neurotransmission. It is shown in animal models that the neuronal content of enkephalin peptides is reduced by chronic activation of dopaminerg neurotransmission [10]. Pain sensitivity is affected by the neuronal content of enkephalin, and reduction in the enkephalin content is shown to be followed by an upregulation of mu opioid receptors [11]. Taken together, this can explain the influence from variation in the *COMT *gene on the effect of opioids in pain treatment.

We also observed that carriers of alleles for six of the SNPs analysed, the Rs5746849 and Rs740603 polymorphism in intron 1, the Rs6269 polymorphism in intron 2, the Rs2239393 polymorphism in intron 3 and the Rs4818 and Rs4680 (Val158Met) polymorphisms in exon 4 were weakly associated to morphine dose (Table 3). The alleles associated with the lowest morphine dose requirements constitute part of the SNP sequence in haplotype 1, which seems reasonable since haplotype 1 is associated with lower morphine dose requirements in this patient cohort. The SNPs defining a haplotype may have functional effects on a protein if the amino acid code is changed [4], and synonymous SNPs may have effects on the secondary structure of mRNA [12], that could alter mRNA stability and/or the translation of a protein [13]. SNPs may also be associated to a phenotype without having any effects neither on the protein nor the mRNA, if it is closely linked to another SNP exerting the real effect on the protein or mRNA. The exact contribution from each SNP in haplotype 1 to the observed effect on morphine requirements in the present study is not known.

In the paper we have constructed long haplotypes across the entire *COMT *gene. An alternative approach would have been to construct haplotypes defined by haploblock boundaries. The latter approach is based on including only SNPs that have a very high probability of being inherited together (visualized by the value of D' or r^2 ^which are correlation factors between SNPs) and as a consequence limiting the gene distance to which SNPs categorize into haplotypes. According to literature the *COMT *gene consists of at least three haploblocks in Caucasians [3,14] and there is consistency between ethnic groups, so the haploblocks is likely to be present also in a Norwegian population. The division of genes into haploblocks limits the number of haplotypes present in the population and thereby increases the number of individuals that fall into each different haplotype group. When analysing long haplotypes across the entire gene fewer individuals in the population will be carriers, but more information will be gained from the effect of combination of SNPs and in that sense long haplotypes may be more biologically relevant. Any sizes of haplotypes will be of more scientifically interests than analyses of SNPs considered one by one.

A cancer population is a heterogeneous group and prone to be influenced from several possible confounders such as severity of disease, organ dysfunction and treatment of other drugs. Therefore, we analysed for possible confounding factors that could influence the need for morphine in cancer pain. We found no differences between carriers and non-carriers of haplotype 1 for patients' symptoms or for patients' demographics, except from the time since diagnosis. There was a tendency that carriers of haplotype 1 have had a cancer diagnosis for a longer time than non-carriers of the haplotype (Table 6). Theoretically, patients with a diagnosis for a long time (that is the patients carrying the haplotype 1) should need more morphine due to more advance cancer disease. In our cohort the carriers of haplotype 1 need less morphine than non-carriers. Thus, a potential bias from the skewed distribution of time since cancer diagnosis is that the observed difference between haplotypes is lower that the true difference between haplotypes. However, in order to further explore if time since diagnosis was an independent predictor of morphine dose we included potential confounding factors in a multivariate analysis. This analysis showed that only "haplotype 1" and "time since morphine treatment started" were predictors for morphine dose. Regression analysis is usually linear, where b is the slope of the graph and gives the change in value of one outcome (e.g. morphine dose), per unit change in the other (e.g. months of morphine treatment). In our regression the association is not linear because we used the logarithm (log_10_) of the 24 hour morphine dose as the dependent variable. Therefore, for each month of morphine treatment, the predicted 24 hour morphine dose increases by a factor of 10^(b × months) ^which translates to that the dose on average increases by 43% every 12 months (10^0.013 × 12^). Patient carrying haplotype 1 is predicted to need less morphine to relieve pain than a patient not carrying haplotype 1, with a reduction factor of 10^(b) ^= 10^(-0.147) ^= 0.713. In other words, if a patient, not carrying haplotype 1 need 100 mg of morphine to relieve pain, a patient carrying haplotype 1 is predicted to need 71 mg of morphine to relieve similar pain. The difference we observe in the median morphine dose between non-carriers and carriers of haplotype 1 is of similar order of magnitude, 100 mg versus 60 mg of the 24 hour morphine dose respectively (Table 4a). Experimental studies including healthy volunteers give more controlled experimental conditions due to less potential confounders. However, clinical studies including cancer patients, such as this study and the study by Ross *et al*.,[9] are needed to observe if genetic variability do influence morphine treatment in the patients actually receiving the drug. The best effort in a clinical population is therefore to include potential confounders in the analyses and interpret findings within the clinical context.

Ross *et al*., analysed the *COMT *gene and its association with the central side effects of morphine in a cancer patient cohort. They found that a haplotype present in 10.4% of the population was associated to drowsiness and confusion or hallucination [9]. SNPs in the promoter region and in the intron 1 region define this haplotype and the authors suggest that it is this region of the *COMT *gene that is of interest in order to explain clinical effect from the COMT enzyme. Alterations in the promoter and intronic region of the gene can influence the regulation of gene expression. Therefore, polymorphisms in these regions might be as important as functional SNPs in coding regions. The Ross study did not find any associations between the Rs4680 (Val158Met) polymorphism and central side effects of morphine [9]. Haplotype 1 in the present study is not identical to the haplotype that Ross and co-authors observed to be associated to central side effects of morphine. However, the haplotypes identified as important by Ross *et al*., and haplotype 1 in our study are related as 7 of 10 possible SNP positions from the Rs5746849 polymorphism in intron 1 to the UTR' region carry the same allele and both haplotypes carry the A allele at the Rs4680 (Val158Met) polymorphism. An explanation for the discrepancy of the haplotypes might be that efficacy for pain relief and risks of adverse effects have different relationships to genotypes.

The need for morphine is a result of both the efficacy of morphine and influenced by the patients' pain perception. Patients can experience variable pain from a given nociceptive stimuli. Therefore genetic variability related to opioid efficacy as studied in the present study is closely linked to genetic variability related to pain perception.

Diatchenko *et al*., have investigated *COMT *gene variability and association to pain responses [3]. They identified three haplotypes in the *COMT *gene strongly associated to pain sensitivity and they designated the different haplotypes as low pain sensitivity (LPS), average pain sensitivity (APS) and high pain sensitivity (HPS) haplotypes. Four SNPs (Rs6269, Rs4633, Rs4818 and Rs4680) constitute these haplotypes, of which three of the SNPs (Rs6269, Rs4818 and Rs4680) are included in our analyses. However, Diatchenko *et al*., did not include the region in intron 1 or the promoter regions, the region which the study by Ross and co-authors [9] believe to be the functional region of interest in the *COMT *gene. A direct comparison with our study is difficult because we have included 11 SNPs in our haplotype analyses while Diatchenko *et al*., focused on four SNPs. Kim *et al*., have also investigated *COMT *gene variability and association to pain responses and found that the Rs740603 SNP was associated with maximum post-operative ratings of pain. Even though the comparison between Diatchenko *et al*., [3] and Kim *et al*., [8] with our findings is important, it is also complicated because we investigate the morphine efficacy while they are studying the genetics of pain sensitivity. However, one agreement between the different studies is that the Rs4680 (Val158Met) polymorphism is not the sole explanation of why COMT seem to contribute to the effect on pain perception or opioid efficacy as first reported by Zubieta *et al*., [1] and Rakvag *et al*., [2], respectively.

In the present study the serum concentrations of morphine, morphine-6-glucuoride (M6G) and morphine-3-glucuronide (M3G) reflected the different morphine doses between haplotypes (Table 4b), but did not reach statistical significance as seen for the morphine dose. The interindividual variation of serum concentrations is more pronounced than for the morphine doses. Consequently, a larger effect size or an increased number of patients are needed to reach a statistical significance for an observed difference between serum concentrations.

Numerous SNPs have been detected in the *COMT *gene and 22 of the most frequent SNPs have been analysed regarding different aspects of pain and opioid responses [2,3,8,9], so the analyses of 11 SNPs in our study do not cover all genetic variation in the *COMT *gene. However, as many SNPs are tightly linked within haploblocks, most genetic variability is captured if the selections of SNPs are chosen to represent the different haploblocks building the entire gene [15], which is done in the present study.

In the present study we have carried out several comparisons. Multiple test correction, as the Bonferroni, is used when tests are independent and is therefore highly conservative. In a genetic association study where SNPs usually are partly linked to each other, as is the case for the *COMT *gene, a conservative multiple test correction lead to missing real differences [16,17]. Also, in our study the haplotype analyses is the primary outcome and then as a consequence we do not consider all null hypotheses to be of equally importance [17]. In addition to the haplotype analyses, differences at genotype level and allele level are presented in this study, but differences at 0.01 < p < 0.05 are interpreted with caution and reported as weak associations between genetic groups.

## Conclusion

This study suggest that genetic variability in the *COMT *gene influence the efficacy of morphine in cancer pain patients, and that increased understanding of this variability is reached by expanding from analyses of single SNPs to haplotype constructions and analyses.

## Materials and methods

### Ethics

The study was carried out in accordance to the principles of the Helsinki declaration. The Regional Committee for Medical Research Ethics, Health Region IV, Norway, approved the study. All patients gave their oral and written informed consent before inclusion in the study.

### Subjects

We investigated the same cohort as previously described by Rakvag *et al*., [2]. Two hundred and seven patients were included in the original study, but blood for further genetic analyses was not available for 10 patients. Therefore, 197 patients were available for further genotyping and included in our analyses. All 197 patients were Caucasians, and all received scheduled oral morphine for cancer pain treatment.

### Assessments

Pain was measured using the item "average pain" during the last 24 hours in the Brief Pain Inventory (BPI) questionnaire. The patients rated pain on an 11-point numeric scale, where 0 represents "no pain" and 10 represents "pain as bad as you can imagine". The BPI is developed for the use in cancer pain patients, validated in Norwegian, and recommended by the European Association of Palliative Care for use in clinical studies [18-20]. The European Organization for Research and Treatment of Cancer core quality-of-life questionnaire (EORTC QLQ-C30) version 3.0 was used to assess the patients' nausea/vomiting, constipation, fatigue, sleep, appetite and dyspnea [21]. Cognitive function was assessed with the Mini Mental State (MMS) examination. The MMS score ranges from 0 to 30, higher scores meaning better cognitive function [22]. The patients' functional status was assessed by the Karnofsky performance status [23]. Survival time, time since start of morphine, cancer diagnoses and opioid doses were obtained from the patients' hospital records.

### Blood samples and pharmacogenetic analyses

Collection of blood samples and determination of serum concentration of morphine and its metabolites (morphine-6-glucuronide and morphine-3-glucuronide) were done as described in a previous work from our group [24]. Creatinine serum concentrations and albumin serum concentrations were measured using standard analytical methods.

The genotyping was performed at the Clinical Genomics Group, Imperial College in London, UK. The selection of SNPs for this study and primer sequences for sequence specific polymerase chain reaction (SSP-PCR) are described in a study by Ross and co-authors investigating another cohort and another primary outcome [9]. The selection was based upon frequency of SNP, position in gene and what was known in the literature at the time research was planned. Of the 13 polymorphic SNPs included in Ross and co-authors' study, the rs174680 and the rs7290221 polymorphisms in intron 1, were not analysed in our patient cohort due to very tight linkage with the rs7287550 polymorphism and the rs5746849 polymorphism respectively. As the reaction of the Rs4633 polymorphism did not work very well, we excluded this polymorphism in the present study, but included the Rs4818 polymorphism in exon 4, which had not been analysed in the previous Ross study. Together, 11 SNPs were genotyped in the present study.

Genomic DNA was isolated from 50 to 200 μL EDTA blood on a MagNA Pure LC (Roche Diagnostics Scandinavia AB, Bromma, Sweden) using the MagNA Pure LC DNA Isolation Kit I applying the manufacturers high performance protocol. Purified genomic DNA was eluted in 100 μL antiseptic water and stored at -20°C. Genotypes were determined using sequence specific primers in a polymerase chain reaction (SSP-PCR) [25]. A sequence specific primer and a consensus primer produce a DNA product of known size in this PCR. The sequence specific primer has a mismatch at the 3' end which is designed to identify each genotype variant. The PCR were carried out as described in Ross et al. [26]. PCR products were then electrophoresed on 1.5% agarose gels (Bioline Ltd, London, UK) containing 0.14 mg/mL ethidium bromide (Sigma Ltd, Poole, UK), at 200 volts/cm^2 ^in 0.5% tris borate EDTA buffer (Sigma Ltd, Poole, UK). Products were visualised with a UV illuminator and photographed with a Polaroid camera. The presence of an allele specific band of the expected size, in conjunction with a control band was used to identify an allele.

### Construction of haplotypes

Genotype and allele frequencies and allele carriage were calculated and checked for Hardy-Weinberg equilibrium. Haplotypes were constructed from genotype information from each patient using the computer program Phase [27,28].

### Statistical analyses

The statistical software SPSS for windows v. 14.0 was used to run non-parametric tests and to run a stepwise linear regression analysis. Because the *COMT *alleles are expected to be codominant with respect to the Rs4680 (Val158Met) polymorphism and COMT enzyme activity, we used the Jonckheere-Terpstra test for comparison between genotype groups, working with a hypothesis that μ_1 _≤ μ_2 _≤ μ_3 _(or the opposite μ_1 _≥ μ_2 _≥ μ_3_) [29]. For all other SNPs we used the Kruskal-Wallis test for comparison between genotype groups. We used the logarithm (log_10_) of the 24 hour morphine dose as the dependent variable in the regression analyses because the 24 hour morphine dose, as expected, did not display a normal distribution. The analysis was a stepwise enter linear regression with a criterion for removal of a variable of p > 0.1. The variables included in the regression analysis as independent variables were: haplotype 1, age, gender, tumour diagnosis, Karnofsky performance status, creatinine and albumin serum concentration, time since diagnosis, time since morphine treatment started, survival time after study, BPI average pain score, EORTC score for fatigue, nausea and vomiting, dyspnea, sleep, appetite and constipation, and finally the sum score for the Mini mental examination measuring cognitive function.

Interpretation of p values in this study is done with caution considering the multiplicity of tests carried out.

## Competing interests

The authors declare that they have no competing interests.

## Authors' contributions

PK, SK, FS and TTR conceived of the study and PK, FS and SK collected the experimental data. TTR, JRR and HS carried out the molecular genetics and statistical analysis. All authors drafted the manuscript and approved the final version.
